# Concave Magnetic-Responsive Hydrogel Discs for Enhanced Bioassays

**DOI:** 10.3390/bios14120596

**Published:** 2024-12-05

**Authors:** Amin Ghaffarzadeh Bakhshayesh, Huiyan Li

**Affiliations:** School of Engineering, University of Guelph, Guelph, ON N1G 2W1, Canada; amingb@uoguelph.ca

**Keywords:** concave hydrogel discs, bioassays, magnetic-responsive hydrogels

## Abstract

Receptor-based biosensors often suffer from slow analyte diffusion, leading to extended assay times. Moreover, existing methods to enhance diffusion can be complex and costly. In response to this challenge, we presented a rapid and cost-effective technique for fabricating concave magnetic-responsive hydrogel discs (CMDs) by straightforward pipetting directly onto microscope glass slides. This approach enables immediate preparation and customization of hydrogel properties such as porosity, magnetic responsiveness, and embedded particles and is adaptable for use with microarray printers. The concave design increased the surface area by 43% compared to conventional hemispherical hydrogels, enhancing diffusion rates and accelerating reactions. By incorporating superparamagnetic particles, the hydrogels become magnetically responsive, allowing for stirring within reagent droplets using magnets to improve mixing. Our experimental results showed that CMDs dissolved approximately 2.5 times faster than hemispherical ones. Numerical simulations demonstrated up to a 46% improvement in diffusion speed within the hydrogel. Particles with lower diffusion coefficients, like human antibodies, benefited most from the concave design, resulting in faster biosensor responses. The increased surface area and ease of fabrication make our CMDs efficient and adaptable for various biological and biomedical applications, particularly in point-of-care diagnostics where rapid and accurate biomarker detection is critical.

## 1. Introduction

Biosensors utilize biomolecular components to detect and signal the presence or activity of target analytes [1,2]. Because of the critical role of biosensors in detecting and quantifying biological analytes, numerous biosensors have been created using different methods, each with its own unique features. One popular type of biosensors is the receptor-based biosensor, which has attracted significant attention as an emerging technique for chemical sensing [3]. One advantage of this tool is its high substrate selectivity, which comes from the biomacromolecule receptor [4]. Hydrogels can enhance receptor-based biosensors by providing scaffolds that maintain an aqueous environment [5]. Hydrogel sensors are chemical detectors made from polymer structures that respond to stimuli [6]. Hydrogels are cross-linked polymers forming a three-dimensional network structure that can absorb and retain substantial amounts of water while maintaining their structure [7]. In order for point-of-care diagnostics to be effective, biosensor platforms such as hydrogel sensors must be fast, sensitive, and portable to quickly and accurately detect specific biomarkers in biological samples [8]. However, one common limitation of receptor-based biosensors is the slow diffusion of analytes, resulting in prolonged assay times [9]. 

One method to enhance the efficiency of diffusion and biomolecule attachment is to increase the surface area of the sensors [10,11,12,13]. As the contact area expands, the influence of the diffusion coefficient on mixing increases [14]. Consequently, researchers have focused on increasing the surface area to volume ratio of hydrogels. For example, a biocompatible pectin–oligochitosan hydrogel capsule was shaped like a red blood cell to provide a higher surface area to volume ratio, enhancing transport capacity relative to a spherical design [15]. Red blood cells have approximately 40% more surface area than spherical cells of equivalent volume, resulting in enhanced deformability and optimized internal diffusion processes by reducing the dead space present in the cores of spherical cells [16]. 

In recent years, innovative approaches to particle fabrication have demonstrated the advantages of non-spherical geometries for enhanced performance in various applications. For instance, the vortex ring freezing method, which enables the mass production of particles with unique shapes such as teardrops, jellyfish, and donuts, has shown significant potential in bioencapsulation and cell culture due to the improved surface-to-volume ratios of these structures [17]. Another approach, stop-flow lithography, was used to fabricate highly porous, nonspherical particles with tunable pore sizes. These particles exhibited enhanced surface area and optical properties, making them suitable for drug loading and optical sensors [18]. However, these methods to increase surface-to-volume ratios require complex and costly techniques.

**Figure 1:**
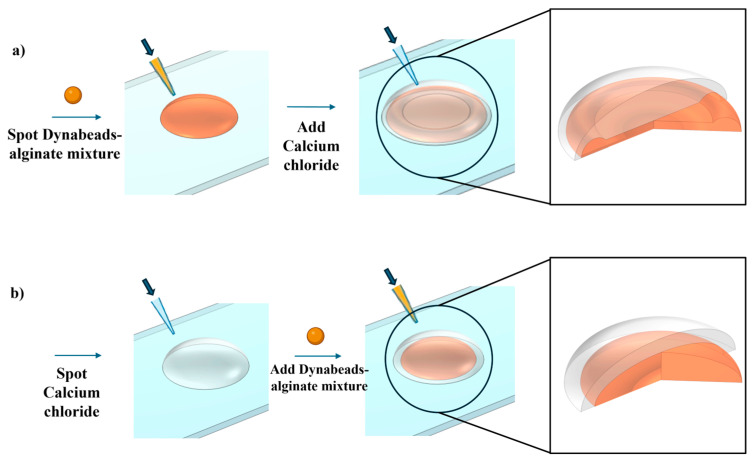
Schematic of the magnetic-responsive hydrogel disc preparation: (**a**) concave hydrogel discs; (**b**) hemispherical hydrogel discs.

To address these challenges, we introduced a fast, simple-to-implement, and cost-effective method to create concave hydrogel discs on-site during a bioassay. In this method, hydrogels form instantly on microscope slides using a simple pipette. This simplicity also allows the approach to be compatible with microarray printers. Specifically, a drop of a cross-linker like calcium chloride is added to the centre of an equal volume of polymer such as alginic acid, using simple pipetting techniques to form concave hydrogel discs, as depicted in Figure 1a. This approach, unlike previous studies, does not require any special instruments or conditions, making it feasible to perform in any laboratory with basic setups. Most importantly, unlike mass-produced concave hydrogels, the properties of the hydrogel, such as porosity, magnetic responsiveness, and the embedded particles, can be easily adjusted by end users. 

In the second part of our study, we presented a technique to increase the speed of the assay by incorporating magnetic particles into concave hydrogel discs to fabricate magnetic-responsive hydrogel (CMDs).

Incorporating magnetic particles into hydrogels can significantly enhance the sensitivity and efficiency of detection [19]. For example, magnetic-responsive hydrogels have been used to fabricate magnetically driven micromotors for detecting DNA from apoptotic tumour cells [20]. 

In our technique, we stirred CMDs within the drop of reagents by using simple magnets to increase the speed of the biochemical reaction. This technique increases the availability of fast bioassays. Current techniques employing magnetic-responsive hydrogels as biosensors typically require complex and expensive processes. These include droplet-based microfluidic technology [21], off-chip UV exposure [22], and the use of hot-melt dispensers [20]. In contrast, our technique only needs magnetic particles, simple magnets, and a microscope slide. 

## 2. Materials and Methods

At the outset of our study, a series of experimental tests were conducted to obtain the essential geometric dimensions for accurately modelling hydrogel behaviour. These observations served as the foundation for our numerical simulations. Subsequently, a mesh independence study was performed to confirm that the simulation results were unaffected by mesh density. With mesh independence established, simulations of the diffusion behaviour of both concave and hemispherical hydrogel discs were carried out to compare diffusion rates between these two geometries. Finally, the numerical results were compared with experimental data on the dissolution rates of concave and hemispherical hydrogel discs in an EDTA solution to evaluate the performance of CMDs produced using this rapid and cost-effective fabrication method.

### 2.1. Experimental Methods

We prepared a 2 wt% alginate solution by dissolving 0.1 g of alginic acid sodium salt from brown algae (low viscosity; Sigma-Aldrich, Saint Louis, MO, USA, Catalog No. A1112) in 5 mL of Milli-Q water (resistivity 18.2 MΩ·cm at 25 °C), stirring the mixture overnight at room temperature for complete dissolution. Subsequently, we added sodium chloride (NaCl, Fisher Chemical, Pittsburgh, PA, USA, Catalog No. S271) to achieve a final concentration of 0.15 M. Invitrogen Dynabeads Protein A (diameter 2.8 µm, 30 mg/mL; Thermo Fisher Scientific, Pittsburgh, PA, USA, Catalog No. 10001D) were vortexed for 30 s to resuspend the stock, and 20 µL of the resuspended beads were transferred to a fresh tube. The tube was placed on a magnet to separate the Dynabeads from the supernatant, which was subsequently removed. Next, 5 µL of the Dynabeads suspension was added to 15 µL of the prepared alginate solution, resulting in a Dynabeads–alginate mixture. 

We then deposited 0.5 µL of the mixture onto a polystyrene microscope slide using a micropipette, as illustrated in Figure 1a. To form concave hydrogel, a 0.5 µL drop of 0.1 M calcium chloride (CaCl_2_, anhydrous, powder, Sigma-Aldrich, Saint Louis, MO, USA, Catalog No. C4901) solution was added to each Dynabeads–alginate spot. The slides were then incubated for 10 min to allow for complete gelation of the hydrogel discs. After gelation, the slides were placed on magnets to immobilize the hydrogels. The hydrogels were then gently washed with Milli-Q water to remove any unreacted materials, and excess water was carefully removed by pipetting. As a result, we obtained the final CMDs. To fabricate hemispherical hydrogel structures, initially, 0.5 µL of Dynabeads–alginate mixture was precisely deposited onto each 0.1 M CaCl₂ spot using a micropipette, as depicted in Figure 1b. Subsequently, 0.5 µL of the Dynabeads–alginate mixture was carefully layered on top of the CaCl_2_ droplet. Cylindrical magnets with dimensions of 2 mm in diameter and 3 mm in height were used to immobilize and move the magnetic-responsive hydrogel discs on microscope slides. 

To compare the dissolution rates of concave and hemispherical magnetic-responsive hydrogel discs, the discs were placed at a distance of 2 mm from a rectangular magnet measuring 12 mm × 6 mm × 2 mm. Then, 2 µL of 0.1 M ethylenediaminetetraacetic acid and disodium salt dihydrate (EDTA, Fisher Chemical, Catalog No. S311) was added to each hydrogel disc, and the dissolution process, along with the movement of the magnetic particles toward the rectangular magnet, was recorded using a camera.

### 2.2. Numerical Methods

**Figure 2:**
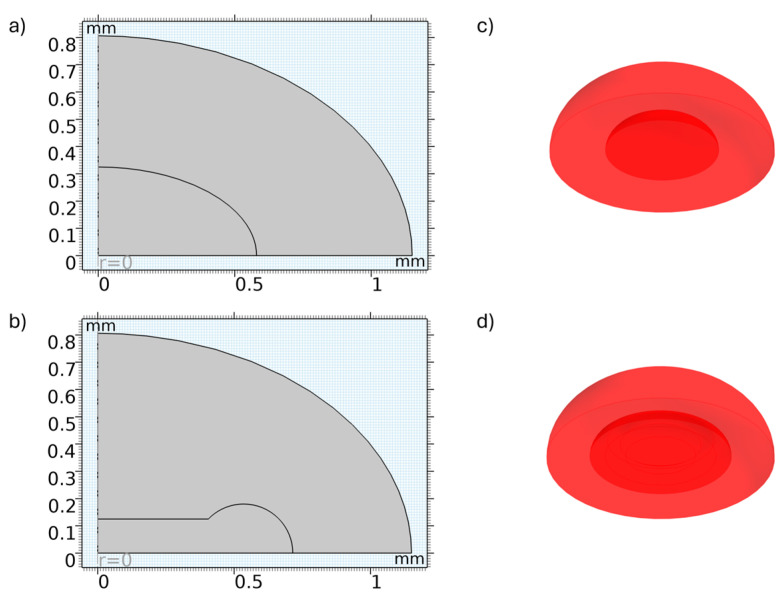
Hydrogel disc geometries and revolved models in the numerical simulation: (**a**) 2D axisymmetric geometry of the hemispherical hydrogel disc; (**b**) 2D axisymmetric geometry of the concave hydrogel disc; (**c**) 3D visualization of the hemispherical disc obtained by revolving the 2D geometry; (**d**) 3D visualization of the concave hydrogel disc obtained by revolving the 2D geometry.

A time-dependent numerical model was developed using COMSOL’s transport of diluted species module to simulate particle movement from droplets to hydrogel discs. A triangular mesh was employed to discretize the geometry. It was assumed that both the hydrogel and the droplet exhibit axisymmetric shapes, allowing the modelling to be performed in a two-dimensional axisymmetric space. After computation, three-dimensional plots were generated by revolving the two-dimensional dataset, as depicted in Figure 2. This approach significantly reduces computational time and resource requirements.

#### Geometry and Boundary Conditions

Experimental images of the hydrogel discs were utilized to define their geometric parameters for the numerical simulations. We employed a Nikon Eclipse Ti microscope equipped with a Nikon DS-Qi1Mc monochrome digital camera with a 4× lens. Images of the hydrogels were captured at various time points and stages of drying. Based on these observations, simulation models utilized an average diameter value derived from the microscopic measurements.

The geometry of the hydrogel discs was modelled based on the assumption of water loss during the cross-linking process. As reported by Łabowska et al. [23] in their study on alginate hydrogels, cross-linking significantly affects the water retention and dimensional stability of hydrogels. According to this study, 2 wt% alginate-based hydrogel beads exhibited a 54.19% water loss during the drying process at 7 °C and 50% humidity, after 10 min of cross-linking with a 0.1 M calcium chloride solution. We used these findings to estimate the final volume of the hydrogel discs in our simulations. Since our experimental setup employed similar cross-linking conditions, we assumed that the initial volume was reduced by 54.19%.

The diffusion coefficient of lysozyme in 2 wt% alginate hydrogel, with a hydrodynamic radius of 2 nm, was taken as 3.17 × 10^−11^ m^2^/s [24]. The diffusion coefficients of other particles in alginate hydrogels were estimated using the effective diffusion coefficient model as described by Oyaas et al. [25]. The study found that, for the nine solutes tested, the effective diffusion coefficients in 2% calcium alginate gel beads were approximately 85% of their diffusivity values measured in water.

The diffusion coefficients for different particles in water and alginate hydrogel are summarized in Table 1. The values for alginate hydrogel were calculated using an effective diffusion coefficient model where the diffusion coefficient in the alginate hydrogel was assumed to be 85% of the corresponding value in water. It is worth noting that Secretory IgA (S-IgA) is formed when two or three IgA molecules are connected by a stabilizing J chain secreted by plasma cells, along with a secretory component produced by mucosal epithelial cells [26]. The S-IgA samples analyzed for Table 1 showed substantial aggregation. Consistently, polyacrylamide/agarose gel electrophoresis of these preparations revealed a broad protein band ranging from 400,000 to 4,000,000 Da [27]. In the numerical model, we set the initial concentration of proteins in the surrounding solution to 1 mol/m^3^, while the initial concentration within the hydrogels was zero.

## 3. Results and Discussion

To illustrate the advantages of our CMDs, we experimentally compared the dissolution rates of CMD and hemispherical magnetic-responsive hydrogel discs in an EDTA solution. Additionally, we conducted numerical simulations to analyze the diffusion of various particles within concave and hemispherical magnetic-responsive hydrogel discs. 

**Figure 3:**
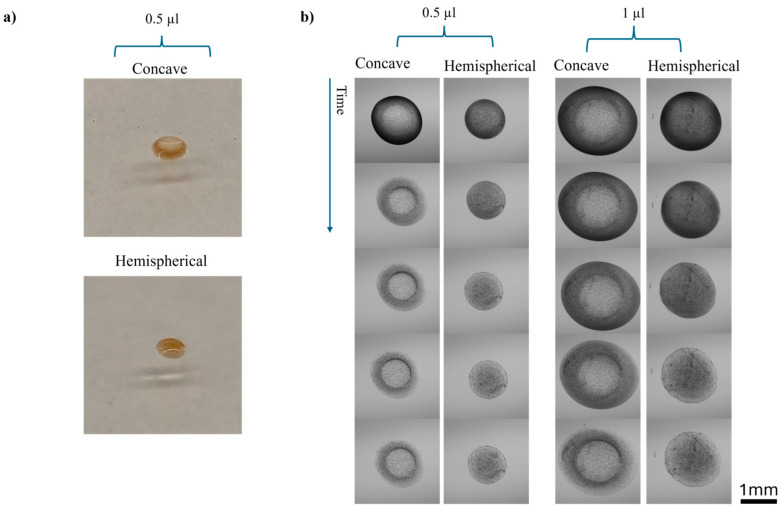
Images of the magnetic-responsive hydrogel disc: (**a**) side-view images; (**b**) time-lapse comparison of drying behaviour in concave and hemispherical magnetic-responsive hydrogel discs. Monochrome images were taken every 10 min.

**Figure 4:**
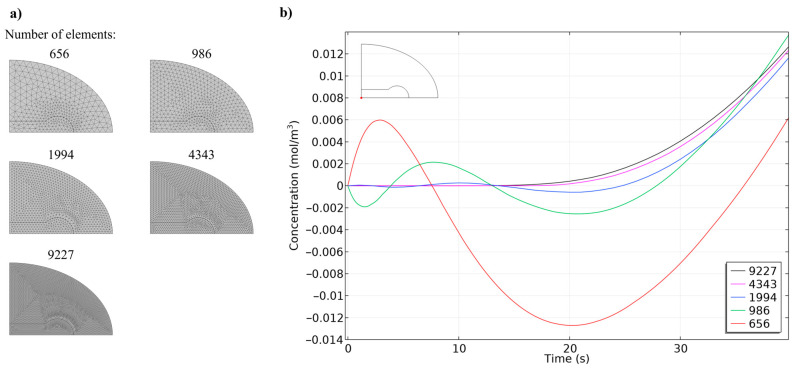
Mesh refinement on the computational model: (**a**) meshed computational domain of the developed model for different numbers of elements; (**b**) concentration of lysozyme in the centre of concave discs’ base (red point) for different numbers of elements.

**Figure 5:**
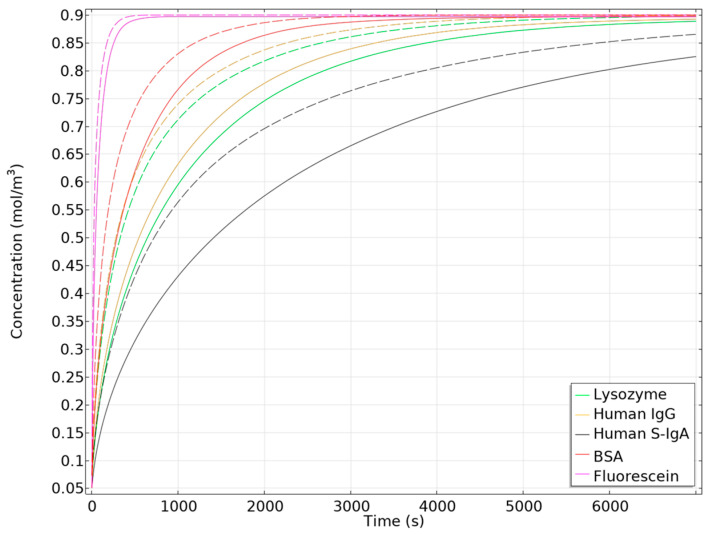
Concentration of different bioparticles in concave (dashed line) and hemispherical hydrogel discs (solid line) at different times in numerical models.

**Figure 6:**
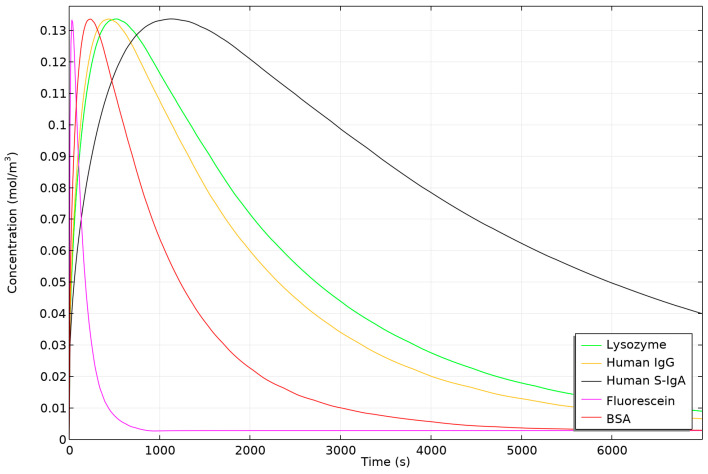
Relative concentration changes of particles in concave versus hemispherical hydrogel discs in numerical models.

**Figure 7:**
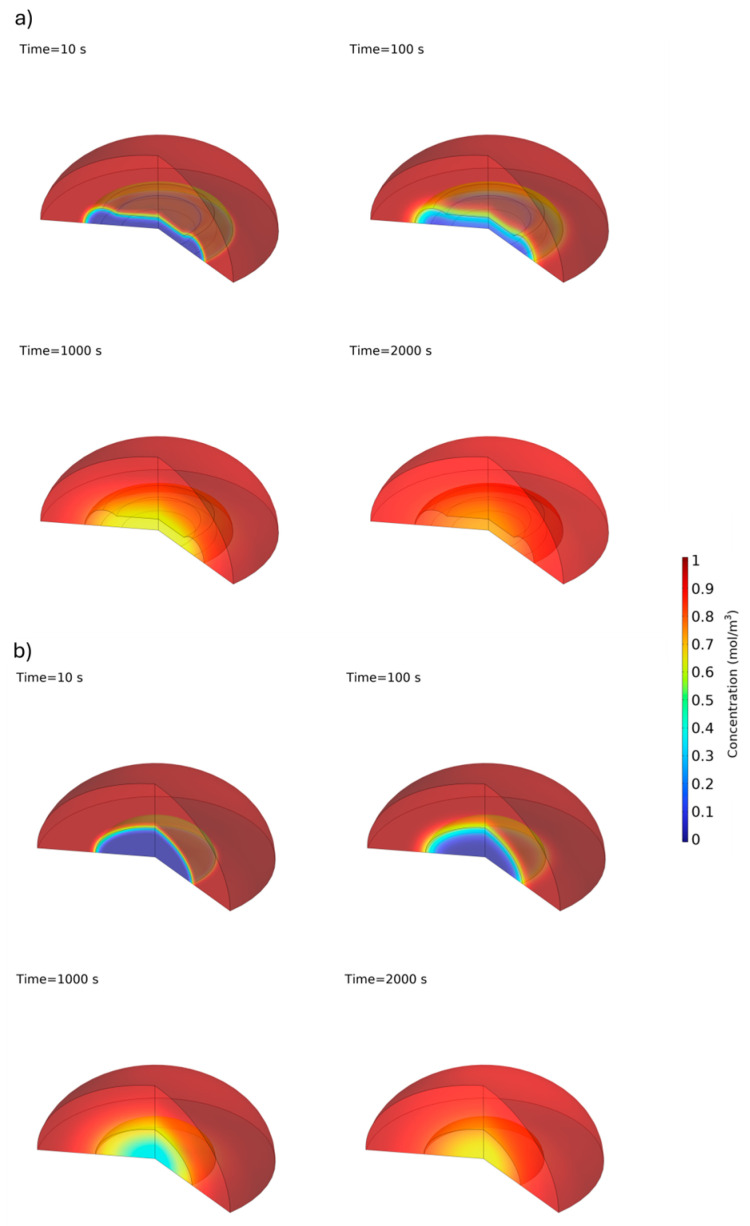
Concentration of lysozyme in concave (**a**) and hemispherical (**b**) hydrogel discs and droplets at t = 10 s, 100 s, 1000 s, and 2000 s in numerical model.

**Figure 8:**
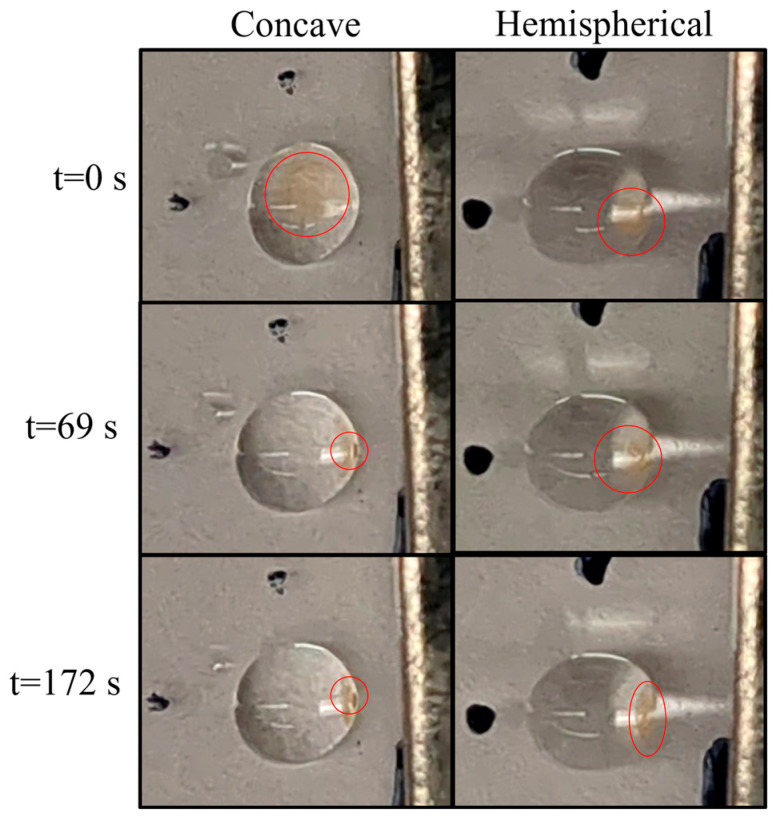
Comparison of the dissolution of concave and hemispherical magnetic-responsive hydrogel discs in an EDTA solution over time. The gel–bead regions are circled.

To accurately characterize the dimensions for our numerical simulations, we captured microscopic images of both concave and hemispherical hydrogel discs at various drying stages, as shown in Figure 3. Subsequently, we conducted a mesh independence study (Figure 4) using five different mesh configurations to ensure the reliability and accuracy of our simulations. Once mesh independence was established, we performed a time-dependent simulation over 7000 s. We then compared the diffusion behaviours of concave and hemispherical hydrogel discs, as depicted in Figure 5, Figure 6 and Figure 7 and Video S1. The numerical results highlighted significant differences in diffusion rates between the two hydrogel geometries. Finally, we compared the numerical findings with experimental results on the dissolution of concave and hemispherical hydrogel discs in EDTA solution (Figure 8). This comparison demonstrated the practical advantages of CMDs produced using this rapid and cost-effective fabrication method towards biosensing applications.

Figure 3 depicts concave and hemispherical magnetic-responsive hydrogel discs produced using the method explained in Figure 1. When the calcium chloride solution is added onto the alginate mixture (Figure 1a), cross-linking initiates at the top surface and progresses downward. The immediate cross-linking at the interface, combined with surface tension effects and the downward momentum of the top droplet due to gravity, pulls the hydrogel edges upward, forming a concave shape. Specifically, the polymerization at the intersection of the two droplets forms a layer between them. Due to the downward movement of this layer during polymerization, liquid is pulled from the centre of the drop to the edges. As polymerization completes at the sides, the edges become thicker than the centre. This is evidenced by the higher concentration of Dynabeads at the outer parts of the hydrogel, as shown in Figure 3.

In contrast, when the alginate mixture is added onto the calcium chloride solution (Figure 1b), cross-linking begins from the bottom upward due to the upward diffusion of calcium ions. This results in a hemispherical shape because the gel material accumulates at the base.

Figure 3b presents a time-lapse comparison of the drying behaviour of concave and hemispherical hydrogel discs. Analysis of these monochrome images revealed that the diameter of both hydrogel types remained relatively constant throughout the drying process, indicating that the diameter can be considered approximately constant. This behaviour is likely due to the hydrogel’s adhesion to the polystyrene microscope slide. Consequently, any significant dimensional changes during drying are attributed to variations in thickness, suggesting that the drying process primarily affects the vertical dimension of the hydrogels. The consistent diameter simplifies geometric modelling in our simulations, allowing us to focus on thickness changes to represent the hydrogels’ physical behaviour during drying, as supported by Łabowska’s study [23]. Accordingly, the hydrogel volume reduces to approximately 54.19% of the initial 0.5 µL volume. We determined the surface areas by constructing detailed 3D models using COMSOL Multiphysics 5.1 software, based on the experimentally measured diameters presented in Figure 3 and the final volumes after accounting for water loss during cross-linking and drying. We calculated the necessary thicknesses to achieve the final volume in COMSOL. Using these 3D models, we employed COMSOL to calculate the total surface areas. Our calculations showed that although both the concave and hemispherical hydrogels had the same volume, the total surface area of the CMD was 3.7 mm^2^, whereas that of the hemispherical hydrogel was 2.6 mm^2^, indicating that the CMD has a surface area approximately 43% greater than that of the hemispherical hydrogel.

Moreover, when considering the surface area in contact with a particle-containing solution droplet, CMDs exhibited an interface area of 2.1 mm^2^, which was 37% larger than the hemispherical hydrogel’s interface area of 1.5 mm^2^. Our findings indicated that CMD offers a significantly larger surface area for interactions with the surrounding solution.

In the context of this study, mesh independence was verified by conducting simulations using various mesh sizes to assess the effect of mesh density on the concentration results. Initially, as illustrated in Figure 4, we created a mesh using free triangular elements to achieve an approximately uniform distribution throughout the model. Then, we roughly doubled the number of elements at each step by decreasing both the maximum and minimum element sizes. The mesh configurations used in the study are summarized in Table 2. Figure 4 illustrates the meshed computational domain of the developed model and the concentration of lysozyme at the centre of the concave discs’ base for different numbers of elements (656, 986, 1994, 4343, and 9227 elements). The graph shows that as the number of mesh elements increases, the concentration remains relatively constant between the finer mesh sizes (4343 and 9227). This indicates that further refinement of the mesh beyond 4343 elements does not significantly alter the results, demonstrating that the model has achieved mesh independence. Thus, the mesh with 4343 elements was selected for the subsequent simulations as a balance between computational efficiency and accuracy. The reason Figure 4 focused on the initial times is that the concentration gradient is at its maximum during the initial seconds. This is because the particle concentration at time zero was assumed to be zero in the hydrogel and 1 mol/m^3^ in the solution. As a result, achieving convergence among the solutions from different meshes is more challenging. 

The main purpose of this study is to demonstrate that our proposed procedure significantly increases the surface-to-volume ratio of hydrogels, leading to improved diffusion rates and reaction speeds, crucial for biosensor performance. Figure 5 compares the concentration profiles of various bioparticles with different sizes (lysozyme, Human IgG, Human S-IgA, BSA, and Fluorescein) within concave and hemispherical hydrogel discs in numerical models over time when exposed to a 2 µL solution containing 1 mol/m^3^ of bioparticles. As depicted, the concentration of bioparticles in the concave hydrogel increases faster than that in the hemispherical design. This increase is a direct consequence of the concave hydrogel’s higher surface-to-volume ratio, which enhances molecular diffusion. 

Figure 6 illustrates the relative concentration changes of various particles within concave versus hemispherical hydrogels in numerical models. Despite variations in diffusion coefficients and particle sizes, all examined particles exhibit a consistent maximum concentration difference of 0.13 mol/m^3^ between the two hydrogel geometries. Specifically, lysozyme reaches its peak concentration difference at 520 s, Human IgG at 440 s, and Human S-IgA at 1130 s. Bovine Serum Albumin (BSA) shows the maximum difference much earlier, at 230 s, while Fluorescein attains its peak difference in just 30 s. These results indicate that larger particles with lower diffusion coefficients require more time for the concentration differences between concave and hemispherical hydrogels to stabilize and reach their minimum. This demonstrates that particles with lower diffusion coefficients benefit the most from the concave hydrogel design, leading to enhanced diffusion and faster biosensor response times.

Figure 7 shows the simulated concentration distribution of lysozyme within the concave and hemispherical hydrogel discs and surrounding droplet at four time points: 10 s, 100 s, 1000 s, and 2000 s. Initially, at 10 s, the concentration is primarily concentrated at the disc’s surface, indicating the early diffusion phase. As time progresses, the concentration spreads more uniformly within the hydrogel, with significant penetration observed by 1000 s. After 2000 s, lysozyme is nearly uniformly distributed throughout the CMD, unlike in hemispherical hydrogel discs, where such distribution is not achieved within the same time frame. Video S1 illustrates this distribution for both types of hydrogel discs over the entire 7000 s period. As shown in the video, changes occur more rapidly during the initial seconds because the concentration difference between the hydrogel and the solution is greatest at t = 0 s. 

According to Video S2, it is also possible to move the CMDs within a drop of solution by moving the microscope slide. Moving the hydrogel enhances diffusion and accelerates the reaction in two ways. First, it exposes the base surface of the CMDs to analytes, allowing the solution to contact this surface. Second, it increases the fluid velocity around the hydrogel, thereby raising the Reynolds number; a higher Reynolds number enhances mixing [14]. Therefore, converting the concave hydrogel into a concave magnetic-responsive hydrogel disc further improves the performance of this hydrogel disc shape for conducting bioassays. 

In bioassays, dissolution of the hydrogel can help release captured analytes for downstream analysis. To experimentally compare the dissolution rates of concave and hemispherical hydrogel discs, both hydrogel discs encapsulated with magnetic beads were first formed and subsequently washed with 2 µL of water. Following the washing step, 2 µL of 0.1 M EDTA was added to each hydrogel droplet, ensuring that both were maintained at the same 2 mm distance from the rectangular magnet. As depicted in Figure 8 and Video S3, the CMD began to dissolve at 69 s post-EDTA addition, whereas the hemispherical hydrogel remained intact until 172 s. This indicates that the CMD initiates dissolution approximately 2.5 times faster than the hemispherical hydrogel. These results highlight the enhanced reactivity of CMDs in the presence of EDTA compared to their hemispherical counterpart.

## 4. Conclusions

This study introduces a rapid and cost-effective method for fabricating concave magnetic-responsive hydrogel discs directly within a basic laboratory setting and demonstrates its advantages for conducting rapid bioassays through both experimental data and numerical simulations. By employing simple pipetting techniques to deposit a drop of cross-linker onto a polymer mixture embedded with superparamagnetic particles, we have created hydrogels that are immediately usable and customizable in terms of porosity and magnetic responsiveness. 

Our concave design of the hydrogel discs results in a surface area 43% greater than that of traditional hemispherical hydrogels. This increased surface area significantly enhances diffusion rates and reaction speeds, which are critical factors in biosensor performance. Numerical simulations demonstrated up to a 46% improvement in the speed of antibody diffusion within the concave hydrogels compared to hemispherical designs. Specifically, particles with lower diffusion coefficients, such as larger biomolecules, benefit the most from the concave geometry, leading to faster biosensor response times.

The experimental results further validated the advantages of our CMDs. When exposed to EDTA, CMDs began to dissolve approximately 2.5 times faster than their hemispherical counterparts, initiating dissolution at 69 s compared to 172 s. This rapid dissolution is an indication of the enhanced reactivity of our concave design.

Furthermore, incorporating magnetic particles into the hydrogels not only makes the hydrogels magnetically responsive but also enables stirring within reagent droplets using common magnets. This magnetic responsiveness enhances mixing and exposes more surface area to the surrounding solution. We believe that our CMDs hold great promise for rapid bioassay applications, contributing to the advancement of point-of-care diagnostics and other biomedical fields where quick and accurate detection of specific biomarkers is essential. Future work could involve immobilizing proteins onto the magnetic beads embedded within the hydrogel discs to explore specific binding interactions, such as antigen–antibody pairs. Investigating how the hydrogel geometry affects binding efficiency and specificity would provide valuable insights into the optimization of biosensor designs.

## Figures and Tables

**Table 1:** Diffusion coefficients of different particles in water and alginate hydrogel.

Particle	Molecular Mass (Da) [27]	Diffusion Coefficient in Water (m^2^/s) [27]	Diffusion Coefficient in 2% Alginate Hydrogel Beads (m^2^/s)
Fluorescein	300	5.7 × 10^−6^	4.84 × 10^−6^
BSA	68,000	8.3 × 10^−7^	7.05 × 10^−7^
Human IgA	150,000	5.2 × 10^−7^	4.42 × 10^−7^
Human IgG	150,000	4.4 × 10^−7^	3.74 × 10^−7^
Human IgM	970,000	3.2 × 10^−7^	2.72 × 10^−7^
Human S-IgA *	_	1.7 × 10^−7^	1.44 × 10^−7^

* Aggregates formed by 400,000-Da S-IgA monomers [27].

**Table 2:** Mesh properties used in the mesh independence study.

	Number of Elements	Maximum Element Size (µm)	Minimum Element Size (µm)
1	656	80	30
2	986	50	20
3	1994	33	10
4	4343	22	5
5	9227	15	1

## Data Availability

Data is available upon request.

## Supplementary Materials

The following supporting information can be downloaded at: https://www.mdpi.com/article/10.3390/bios14120596/s1, Video S1: Concentration of Lysozyme in concave and hemispherical hydrogel discs and droplets over time; Video S2: Demonstration of moving CMDs within a solution drop by sliding the microscope slide; Video S3: Comparison of the dissolution of concave and hemispherical magnetic-responsive hydrogel discs in EDTA over time.

## References

[1] Kulshreshtha N.M., Shrivastava D., Bisen P.S., Grumezescu A.M. (2017). 14—Contaminant sensors: Nanotechnology-based contaminant sensors. Nanobiosensors.

[2] Goodrum R., Aggarwal R.T., Li H. (2024). Gold-nanoparticle-embedded membrane (GEM) for highly sensitive multiplexed sandwich immunoassays. Sens. Actuators B Chem..

[3] Wu C., Du L., Zou L., Zhao L., Huang L., Wang P. (2014). Recent advances in taste cell- and receptor-based biosensors. Sens. Actuators B Chem..

[4] Nakano S., Konishi H., Morii T., Chenoweth D.M. (2020). Chapter Nine—Receptor-based fluorescent sensors constructed from ribonucleopeptide. Methods in Enzymology.

[5] Sebben D., Strohle G., Roy P.S., Li H. (2023). Gold-nanoparticle-embedded hydrogel droplets with enhanced fluorescence for imaging and quantification of proteins in cells. Microchim. Acta.

[6] Kato S., Takinoue M., Onoe H. Dual-Sensing Mechanical Hydrogel Biosensor Composed by Aptamer Recognition and DNA Logic Gates. Proceedings of the 2024 IEEE 37th International Conference on Micro Electro Mechanical Systems (MEMS).

[7] Kamini, Puri D. (2024). Hydrogel-based drug delivery systems—A review. Polym. Plast. Technol. Mater..

[8] Kocheril P.A., Lenz K.D., Mascareñas D.D.L., Morales-Garcia J.E., Anderson A.S., Mukundan H. (2022). Portable Waveguide-Based Optical Biosensor. Biosensors.

[9] Henriksson A., Neubauer P., Birkholz M. (2022). Dielectrophoresis: An Approach to Increase Sensitivity, Reduce Response Time and to Suppress Nonspecific Binding in Biosensors?. Biosensors.

[10] Liu Y., Chen Z., Xu J. (2024). Recent advances in the microfluidic generation of shape-controllable hydrogel microparticles and their applications. Green Chem. Eng..

[11] Goodrum R., Li H. (2024). Advances in three dimensional metal enhanced fluorescence based biosensors using metal nanomaterial and nano-patterned surfaces. Biotechnol. J..

[12] Aggarwal R.T., Lai L., Li H. (2023). Microarray fabrication techniques for multiplexed bioassay applications. Anal. Biochem..

[13] Momenbeitollahi N., Aggarwal R., Strohle G., Bouriayee A., Li H. (2022). Extracellular Vesicle (EV) Dot Blotting for Multiplexed EV Protein Detection in Complex Biofluids. Anal. Chem..

[14] Rahbarshahlan S., Ghaffarzadeh Bakhshayesh A., Rostamzadeh Khosroshahi A., Aligholami M. (2021). Interface study of the fluids in passive micromixers by altering the geometry of inlets. Microsyst. Technol..

[15] Harvestine J.N., Mikulski B.A., Mahuta K.M., Crouse J.Z., Guo X., Lee J.C., Midelfort K.S., Chen J., Zhang W. (2014). A Novel Red-Blood-Cell-Shaped Pectin-Oligochitosan Hydrogel System. Part. Part. Syst. Charact..

[16] Waeterschoot J., Gosselé W., Lemež Š., Casadevall i Solvas X. (2024). Artificial cells for in vivo biomedical applications through red blood cell biomimicry. Nat. Commun..

[17] An D., Warning A., Yancey K.G., Chang C.-T., Kern V.R., Datta A.K., Steen P.H., Luo D., Ma M. (2016). Mass production of shaped particles through vortex ring freezing. Nat. Commun..

[18] Li M., Joung D., Kozinski J.A., Hwang D.K. (2017). Fabrication of Highly Porous Nonspherical Particles Using Stop-Flow Lithography and the Study of Their Optical Properties. Langmuir.

[19] Wei X., Bian F., Zhang H., Wang H., Zhu Y. (2021). Multiplex assays of bladder cancer protein markers with magnetic structural color hydrogel microcarriers based on microfluidics. Sens. Actuators B Chem..

[20] Qin F., Wu J., Fu D., Feng Y., Gao C., Xie D., Fu S., Liu S., Wilson D.A., Peng F. (2022). Magnetically driven helical hydrogel micromotor for tumor DNA detection. Appl. Mater. Today.

[21] Moharramzadeh F., Seyyed Ebrahimi S.A., Zarghami V., Lalegani Z., Hamawandi B. (2023). Synthesis and Characterization of Hydrogel Droplets Containing Magnetic Nano Particles, in a Microfluidic Flow-Focusing Chip. Gels.

[22] Feng Y., White A.K., Hein J.B., Appel E.A., Fordyce P.M. (2020). MRBLES 2.0: High-throughput generation of chemically functionalized spectrally and magnetically encoded hydrogel beads using a simple single-layer microfluidic device. Microsyst. Nanoeng..

[23] Łabowska M.B., Skrodzka M., Sicińska H., Michalak I., Detyna J. (2023). Influence of Cross-Linking Conditions on Drying Kinetics of Alginate Hydrogel. Gels.

[24] Kopač T., Krajnc M., Ručigaj A. (2022). Protein release from nanocellulose and alginate hydrogels: The study of adsorption and desorption kinetics. Colloids Surf. B Biointerfaces.

[25] Oyaas J., Storrø I., Svendsen H., Levine D.W. (1995). The effective diffusion coefficient and the distribution constant for small molecules in calcium-alginate gel beads. Biotechnol. Bioeng..

[26] Tosi M.F., Feigin R.D., Cherry J.D., Demmler-Harrison G.J., Kaplan S.L. (2009). Chapter 2—Normal and impaired immunologic responses to infection. Feigin and Cherry's Textbook of Pediatric Infectious Diseases.

[27] Saltzman W.M., Radomsky M.L., Whaley K.J., Cone R.A. (1994). Antibody diffusion in human cervical mucus. Biophys. J..

